# LINEA ALBA COLLAGEN ASSESSMENT IN MORBIDLY OBESE PATIENTS

**DOI:** 10.1590/0102-6720201600S10003

**Published:** 2016

**Authors:** João Vicente Machado GROSSI, Felipe Fernandes NICOLA, Ivan Alberto ZEPEDA, Martina BECKER, Eduardo Neubarth TRINDADE, Vinicius Von DIEMEN, Leandro Totti CAVAZZOLA, Manoel Roberto Maciel TRINDADE

**Affiliations:** Hospital de Clinicas, Federal University of Rio Grande do Sul and Department of Legal Medicine, Instituto Geral de Perícias, Porto Alegre, RS, Brazil.

**Keywords:** Anastomosis, Roux-en-Y, Bariatric surgery, Gastric bypass, Stenosis

## Abstract

**Background:**

The evaluation of collagen in the abdominal wall has been increasingly studied because of the relevance on collagen in the healing process after laparotomy.

**Aim:**

To evaluate the amount of collagen in the linea alba of patients undergoing laparotomic bariatric surgery and comparing with non-obese cadavers.

**Methods:**

Were evaluated 88 samples of aponeurosis from abdominal linea alba of 44 obese patients (obesity group) and 44 non-obese cadavers (control group). The samples were collected in 2013 and 2104, and were sorted according to age (18-30, 31-45 and 46-60), gender, BMI, waist and cervical circumference, and subcutaneous tissue thickness. Material for biopsy was collected from the supraumbilical region of the linea alba for immunohistochemical analysis differentiating collagen type 1 and type 3 and the 1/3 ratio. Image-Pro Plus pixel counting software was used to measure the amount of collagen.

**Results:**

The obesity group evidenced mean age 44.11±9.90 years; 18-30 age group had three (6.8%) obese individuals; 31-45 had 22 (50%) and 46-60 had 19 (43.1%). Females were present in 81.8% (n=36); BMI (kg/m²) was 48.81±6.5; waist circumference (cm) was 136.761±13.55; subcutaneous tissue thickness (cm) 4.873±0.916. Considering age groups, gender and BMI, there were statistical differences in all tests when compared with the cadavers.

**Conclusion:**

The amount of collagen in the linea alba above the umbilical region in the morbidly obese patients was smaller than in the non-obese cadavers in the same age group.

## RESUMO

**Racional:**

A avaliação do colágeno na parede abdominal é cada vez mais estudada, em virtude da relevância dele no processo cicatricial após laparotomia.

**Objetivo:**

Avaliar a quantidade de colágeno na linha alba de pacientes submetidos à cirurgia bariátrica e compará-la com a de cadáveres não-obesos.

**Método:**

Foram avaliados dois grupos com total de 88 amostras da aponeurose da linha alba abdominal, divididas em 44 de pacientes obesos (grupo obesidade) com indicação de cirurgia bariátrica e 44 de cadáveres não-obesos (grupo controle). As amostras foram retiradas da linha alba abdominal no período de 2013 a 2014 e inicialmente foram separadas conforme faixas etárias (18-30, 31-45 e 46-60), gênero, medidas de IMC, circunferência abdominal e cervical e espessura do subcutâneo do indivíduo. Foi coletado material para biópsia da linha alba supraumbilical para análise imunoistoquímica, diferenciando o colágeno tipo I e III e sua relação de tipo I/III. Utilizou-se o programa de contagem de pixels Image-Pro Plus^(r)^, que mensurou a quantidade do colágeno.

**Resultados:**

O grupo obesidade teve idade 44,11±9,90 anos, Na faixa etária de 18-30 anos foram incluídos três (6,8%) obesos; na de 31-45 anos 22 (50%) e na de 46-60 anos 19 (43,1%). O gênero feminino apresentou predomínio, com 36 (81,8%) pacientes. O IMC (kg/m²) foi de 48,81±6,5; a circunferência abdominal (cm) foi de 136,761±13,55; a espessura do subcutâneo (cm) foi de 4,873±0,916. A quantidade de colágeno tipo I foi de 134.683,3±206.657,4; a de colágeno tipo III foi de 413.137,2±283.656,1; a razão do colágeno tipo I/III foi 0,419±0,636. Considerando-se faixas de idade, gênero e IMC, foram constatadas diferenças estatísticas em todas as análises quando comparadas com às dos cadáveres.

**Conclusão:**

Os obesos mórbidos apresentaram quantidade de colágeno na linha alba supraumbilical menor que a do grupo controle de cadáveres não-obesos na mesma faixa etária.

## INTRODUCTION

The repair of abdominal wall hernias remains the most common operation for the general surgeon. For a long time, numerous studies have been developed to search for causes and best method of treatment^1^ ^,^ ^20^. The causes of failure in bringing the abdominal walls together are important research topics^12^ ^,^ ^13^. The incidence of abdominal wall defects in obese patients is high, reaching 30% after laparotomy. Thus, to identify the factors that may influence the degree of strength and resistance of the abdominal wall in patients with high body mass index - above 35 kg/m^2^ - and possible postoperative hernia becomes critical to the surgeon^8^. The replacement of the structural matrix of the abdominal wall from type I collagen by type III has been described as a cause of hernias in the inguinal region^9^ ^,^ ^18^. However, to date, the amount and stratifications have not been established in obese patients and their stratifications in the supra umbilical region.

This study aims to identify the predominant type of collagen in the anterior wall of patients undergoing bariatric surgery, comparing with non-obese cadavers.

## METHODS

Linea alba samples from patients with indication to bariatric surgery and from non-obese cadavers from the Forensic Medicine Department of Porto Alegre were analyzed in the Hospital de Clinicas de Porto Alegre, Porto Alegre, RS, Brazil. The protocol was approved by the Group for Research and Graduate Studies of the Porto Alegre University Hospital and by the Ethics Committee of the Hospital de Clínicas, under protocol number 269331/2013. The study was also approved by the Scientific Division of the Forensic Department of Porto Alegre. 

The study sample consisted of two groups. The number of individuals in each group was stipulated by sample calculation based on the study of Fachinelli, which established the need to measure a minimum difference of 80 pixels between total collagen in obese patients and controls, with a standard deviation of 131 pixels. Considering a statistical power of 80% and a significance level of α = 0.05, at least 44 subjects in each group were needed, totaling 88 individuals to be analyzed. 

The obesity group consisted of 44 patients who underwent gastric bypass surgery with Roux-en-Y in Hospital de Clínicas de Porto Alegre from March 2013 to December 2014. Inclusion criteria were: patients in Digestive Surgery Clinic of the Hospital de Clínicas de Porto Alegre that agreed to the informed consent (IC); patients diagnosed with morbid obesity and to whom bariatric surgery was recommended (BMI>40 or BMI>35 with co-morbidities); older than 18 and younger than 60 years. The exclusion criteria were: patients who underwent prior surgery at linea alba supraumbilical; with lesions or deformities in the front wall; with degenerative diseases; with Marfan syndrome, osteogenisis imperfecta and Ehlers-Danlos syndrome.

The non-obese group consisted of 44 cadavers from the Forensics Department of Porto Alegre. Inclusion criteria were: individuals with up to 12 h of death; older than 18 and younger than 60; with BMI of 18 kg/m^2^ and less than 25 kg/ m^2^. The exclusion criteria were: patients who underwent prior surgery at linea alba supraumbilical; with lesions or deformities in the front wall; with degenerative diseases; with Marfan syndrome, Osteogenisis imperfecta and Ehlers-Danlos syndrome.

The study consisted in the analysis of the collagen in the anterior abdominal wall by laparotomy incision during the procedure with gastrointestinal bypass in Roux-en-Y.

Sample collection of the linea alba sized 1x1 cm was performed at the incision site of the surgical procedure in the midline 5 cm from the angle of the xiphoid process. The sample was stored in formalin solution for fixation and underwent subsequent histochemical study. Immunohistochemistry was performed for staining. Each sample studied underwent two analyses: one slide was used for immunohistochemical staining to detect collagen type I and another slide was stained for collagen type III. A total of 176 slides were stained.^7^

Quantitative and qualitative assessment of collagen types I and III was performed by immunohistochemistry, using the polyclonal anti-collagen type I (PA1-85317) and polyclonal anti-collagen type III (PA1-85314) antibodies. Was used an imaging system composed of an Olympus microscope with a coupled video camera. The video signal was scanned in 32-bit on a personal computer with a resolution of 1280 (horizontal) by 960 (vertical) pixels and 24 million colors. The software used was Q-Capture Pro 5.1. Ten fields were scanned with a 400 times magnification per slide in a total of 1760 digital images in TIFF file extension format. The scanned images were analyzed using the Application Program Image Pro-Plus, version 3.1 (Media Cybernetics, Silverspring, USA). 

### Statistical analysis

Mann-Whitney U test was used for quantitative variables with asymmetrical distribution; for continuous variables with normal distribution, Student's t test was employed. The tests with p<0.05 were considered statistically significant. SPSS (Statistical Package for Social Science) version 22.0 was used for data analysis.

## RESULTS

The study was conducted until all samples for each group were acquired. There were no surgical complications in the group of obese patients due to the collection of tissue sample. The descriptive analysis of each group was presented as mean±standard deviation, with the respective percentages

The initial measurement for sample characterization shows an age profile of the obesity group with mean and standard deviation, in years, of 44.11±9.9. There was a predominance of female, with the presence of 36 (81.8%) individuals with weight in kg, 128.7±23.02, totaling a BMI (kg/m²) of 48.89±6.50. Excess weight (weight prior to surgery - corresponding to BMI = 25 its height) was 62.89±19.13 kg, respectively. The non-obese cadavers showed a male predominance, with 36 (81.8%) cases, mean age and standard deviation of 32.7±7.05, weighing 69.96±6.82 and BMI 23.98±1.17.

With regard to comorbidities in the obesity group during the preoperative examination, only two (4.5%) patients were considered healthy, while 42 (95.5%) out of 44 had at least one comorbidity, especially systemic arterial hypertension in 38 (86.4%) patients, diabetes mellitus in 18 (40.9%) and smoking history. With regard to smoking, 10 (22.72%) patients said they had ceased smoking more than two years earlier, two (4.54%) patients had ceased smoking from a year up to three months prior to surgery and 32 (72.72%) denied ever smoking. Among patients with a history of chronic lung diseases, including asthma, only five (11.4%) were users of nasal corticosteroids at a low dose (<100 mcg a day), and 19 (43.2%) patients had symptoms of sleep apnea. Hypothyroidism was present in nine (20.5%) patients, and the number of pregnancies had mean and standard deviation of 1.48±1.83 per patient. In the control group, patients with a history of comorbidities, including smoking and pregnancy, were excluded.

Anthropometric measurements were used to differentiate the distribution pattern of weight and fat in both groups. The obesity group showed statistically significant measurements in all features, including height with mean and standard deviation of 170.62±7.49 vs 162.36±7.64, in cm, p<0.01 when compared to the non-obese cadavers. Followed by the BMI analysis, with rates of 48.89±6,50 versus 23.98 ±1.17 kg/m², p<0.01. Abdominal circumference measured in cm (136.76±13.55 vs 80.06±6.82), neck circumference (42.57±4.20 vs 36.57±2.05), arm circumference (41.66±4.89 vs 27.33±3.62), waist circumference (121.45±12.16 vs 78.41±6.34) and hip circumference (139.33±15.58 vs 90.26±7.72) had a statistically significant difference of p<0.01 in the obesity and in the non-obese cadavers, respectively. The thickness of subcutaneous fat at the biopsy site for the linea alba in the obesity group presented mean and standard deviation (in cm) of 4.87±0.91 vs 1.94±0.61 in the non-obese cadavers, p=0.007.

**FIGURE 1:**
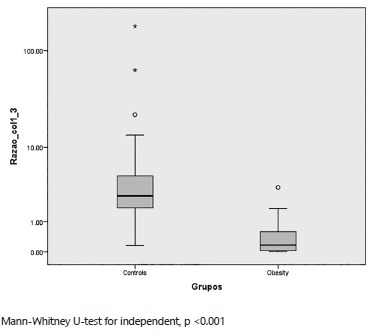
Comparison of obesity vs non-obese cadavers groups in the ratio of collagen type I / III: samples shows significant difference with an increased amount of collagen in the control group compared to obese

The collagen count was done through the Image-Pro Pus(r) program in photographs obtained by microscopy 400x, which captures the image and transforms it into pixels, 1200 by sample field. The obesity group had a mean and standard deviation of 134,683.3±206,657.4 pixels in collagen type I samples and 413,137.2±283,656.1 pixels in collagen type III samples and 0.419±0.636 was the average ratio of collagen type I/III. The control group (non-obese group) had an average quantity±standard deviation of 1,587,378.8±719,527.5 pixels for type I collagen, an average of 630,629.1±396,242 for collagen type III and the ratio of collagen type I/III was 27.49±8.96 pixels. When compared using the Mann-Whitney U test, this showed a statistically significant difference (p<0.001) in samples of collagen type III and the ratio of collagens type I/III and also p=0.03 in the collagen type I. When was compared the obesity and non-obese cadavers, the obesity group had a lower count of collagen type I in three samples; using the Mann-Whitney U test, this showed a statistically significant difference p<0.001 in samples of collagen type III and a ratio of I/III and also p=0.03 in collagen type I (Figure 1).

**FIGURE 2:**
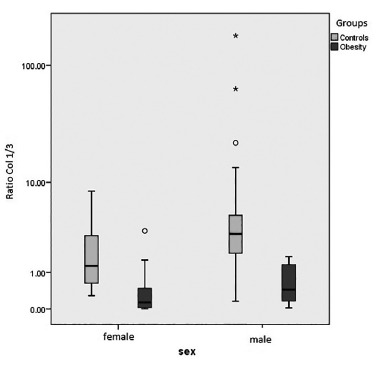
Comparison of obesity and non-obese cadavers groups in the ratio of collagen type I/III distributed by gender

The analysis of collagen per se followed as an attempt to elucidate whether there were differences between samples from patients with the same gender. Comparing the amount of collagen in women, was used the U Mann-Whitney test for independent samples with type III collagen, and the ratio of collagen type I/III presented statistically significant differences, with the highest score in the non-obese group (p<0.001) and collagen type I, p=0.011. In males, there were statistically significant differences only in the collagen type III and the ratio of collagen type I/III, with p<0.01; there was no statistical difference in type I collagen count, with p=0.068 (Figure 2).

**FIGURE 3:**
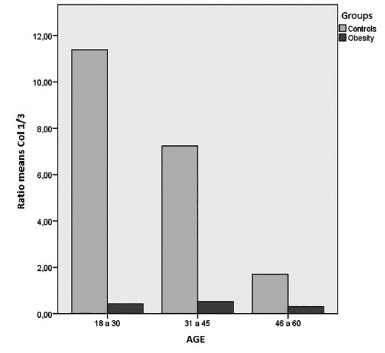
Comparison of age groups according to the ratio of collagen type I/III

Was chosen to divide the samples by age groups for detailed analysis of the collagen. In the first group, with ages ranging from 18 to 30, there were three (6.8%) patients in the obesity group vs 21 (47.7%) in the non-obese cadavers. In the group of 31-45 years old there were 22 (50%) vs 21 (47.7%). In the group 46-60 years, 19 (43.2%) were identified vs two (4.5%) in the non-obese cadavers. Using the Mann-Whitney U test in the analysis of collagen type III and the ratio between I/III in the 18- 30 group, there is a statistically significant difference, with p=0.06, but not in type I collagen, with p=0.965. The same analysis was performed for the other groups, presenting difference in the 31-45 group for collagen type III and the ratio between types I/III, but no statistical difference in the type I collagen analysis, p=0.052. Among older subjects (46-60 years) the same finding about the amount of collagen in the obesity group compared to the cadavers remained with respect to type III collagen and the ratio I/III with p=0.023 and p=0.042, respectively, but there was no evident difference in type I collagen count, with p=0.188. Comorbidities of the patients in the obesity group showed no statistically significant difference when compared to the amounts of type I collagen, type III and the ratio I/III, considering the presence of hypertension, diabetes mellitus, pulmonary obstructive disease and sleep apnea, hypothyroidism, smoking and number of pregnancies (Figure 3).

## DISCUSSION

Collagen continues to be studied to evaluate suitable laparotomy closure and repair of abdominal wall hernias. The study shows a decrease in the total amount and, in particular, a decrease in type I and III collagen and the ratio of type I/III, which may point to a risk factor for wall closure defects, even in situations such as laparoscopy and robotic surgeries.

For patients considered as high risk, due to high BMI, female gender or elder age, closing alternatives have been considered, with reinforcements or techniques such as the use of meshes and, although still under development, stem cells as a reinforcement in special situations such as in the case of patients submitted to bariatric surgery that develop incisional hernia, which can reach 30% when compared to the non-obese population, where it is up to 15%^7^.

In the process of wound closure, there is an increase of collagen type III that develops into type I, with stronger density and structure due to the type of cellular connection. It is therefore important to monitor the obese patient when he/she has a deficit in the amount of collagen and there is still a weight loss goal set for a relatively short period (two years), reaching more than 50% of the excess weight^6^ ^,^ ^17^ ^,^ ^24^.

Several studies^5,11,14,25^ use obesity as an independent risk factor for the development of early and late postoperative complications. Linear regression models show that obesity changes the wound healing process and raises surgical site infection rates. Furthermore, the thickness of the subcutaneous fat appears to influence these rates, supporting our findings that there is a statistically significant difference in the decrease of collagen and increased thickness of the subcutaneous tissue in obese patients as compared to non-obese cadavers.

The study in male cadavers was divided into two broad age groups (18-30 years and 31-60 years), with similar results in collagen composition in linea alba in the supra and infra umbilical regions, compared with the results of the groups in this study. The decrease in the amount of collagen type I and III was observed in the older age group, reinforcing the theory of weakness in the abdominal wall over the years, even in patients that were not distributed according to risk factors^4^. Even in studies that point to a previously existent incisional hernia, age is classified as a risk factor for adverse events in the postoperative period^3^ ^,^ ^16^.

The characteristics of each gender influence the arrangement of anisotropic fiber tensile forces in different directions along the linea alba, and even the healing phase after midline incision. The female gender was described as a risk factor in a study with over 4000 patients, which also included the BMI and thickness of the subcutaneous tissue. In the samples described here, there was a significant difference in the amount of collagen type I and type III and in the ratio I/III when compared to the cases in males and even between obese and non-obese from the same gender, leading to an increased risk of defects in the synthesis of the abdominal wall in morbidly obese women, which is a part of the growing world population^14^.

These considerations reflect a group of high-risk for defects on the wall after a procedure, requiring the search for alternatives to minimize the negative effects. Alternatives like the use of preventive meshes have been under discussion for several years, with favorable and deleterious outcomes from several studies^7,15,23,24,^.

In response to alternatives for proper closure of the abdominal wall in high-risk patients for incisional hernia, ways to provide substrate for the synthesis of the wall should be investigated, which could point to the advent of mesenchymal stem cells. However, there are still no results indicating that they may play a role in safe repair of the abdominal wall or their preventive use^21^. Some limitations of the study can be better evaluated in randomized clinical trials to assess long-term follow-up of patients with the development or not of postoperatively incisional hernias.

## CONCLUSION

Obese patients had an amount of collagen in the umbilical linea alba smaller than the non-obese group when compared to non-obese cadavers in the same age group.
